# Risk of Long-Term Clozapine Medication over Decades for Cardiac Adverse Events Including Heart Failure and Its Pathophysiology: A Japan and China Retrospective Cohort Analysis

**DOI:** 10.3390/medsci14020306

**Published:** 2026-06-11

**Authors:** Ruri Okubo, Nobutomo Yamamoto, Xiaojun Shao, Taku Omori, Jian Xiong, Changhui Liu, Ryo Kato, Masahiko Murata, Tetsuji Kitano, Yuki Ito, Tomoka Oka, Toshiaki Onitsuka, Eishi Motomura, Kaoru Dohi, Gang Zhu, Motohiro Okada

**Affiliations:** 1 Department of Neuropsychiatry, Division of Neuroscience, Graduate School of Medicine, Mie University, Tsu 514-8507, Japan; okubo-r@med.mie-u.ac.jp (R.O.); yamamoto.nobutomo.fe@mail.hosp.go.jp (N.Y.); ryo-kato@med.mie-u.ac.jp (R.K.); murata.masahiko.iw@mail.hosp.go.jp (M.M.); ito-yu@med.mie-u.ac.jp (Y.I.); tomoka-oka@clin.medic.mie-u.ac.jp (T.O.); onitsuka.toshiaki.vc@mail.hosp.go.jp (T.O.); motomura@clin.medic.mie-u.ac.jp (E.M.); 2 Department of Psychiatry, National Hospital Organization Sakakibara Hospital, 777 Sakakibara, Tsu 514-1292, Japan; 3 Department of Psychiatry, The First Affiliated Hospital of China Medical University, Shenyang 110001, China; junxshao@sj-hospital.org (X.S.); jzkn2010@163.com (J.X.); changhuiliu@163.com (C.L.); gzhu@cmu.edu.cn (G.Z.); 4 Department of Cardiology and Nephrology, Graduate School of Medicine, Mie University, Tsu 514-8507, Japan; omori-t@med.mie-u.ac.jp (T.O.); tkitano@med.mie-u.ac.jp (T.K.); dohik@med.mie-u.ac.jp (K.D.); 5 Jinzhou Kangning Hospital, Jinzhou 121019, China; 6 Shenyang Jing’an Mental Health Hospital, Shenyang 110163, China

**Keywords:** clozapine, long-term medication, heart failure, ejection fraction, Japan, China

## Abstract

**Background/Objectives**: Clozapine is the sole antipsychotic approved for treatment-resistant schizophrenia, but it is a double-edged therapeutic option due to various lethal adverse reactions. This study aimed to assess the risk of long-term clozapine medication-induced cardiotoxicity, which has not yet been fully elucidated. **Methods**: This study is a multicenter retrospective cohort study of patients with schizophrenia in Japan and China who received clozapine monotherapy. Cases for which serum NT-proBNP concentration and LVEF derived from echocardiography were available in 2025 were included. In addition, blood examinations, including those administered by the Japanese Clozaril Patient Monitoring Service, were statistically analyzed as independent variables. **Results**: Among a total of 315 cases, including 99 Japanese (clozapine exposure duration: 57.5 ± 4.0 months) and 216 Chinese (208.1 ± 11.0 months) cases, were enrolled. In both Japan and China, age-standardized prevalence of heart failure among patients with prescribed clozapine were higher compared to general population, with odds ratios of 3.2 (95%CI: 1.4–6.4) and 6.9 (95%CI: 3.6–12.0), respectively. The risk factors for stage-B heart failure associated with clozapine were prolonged exposure duration, higher plasma levels of clozapine, and increasing monocytes. Unexpectedly, over 70% of cases with stage-B heart failure associated with clozapine identified in this study did not have metabolic complications. Other than those with cardiomyopathy, myocardial infarction, ileus, or chronic renal failure, no cases with ejection fraction < 50% were observed, suggesting that stage-B heart failure associated with clozapine is speculated to be likely suggestive of HFpEF. **Conclusions**: Traditionally, psychiatry has focused on myocarditis and cardiomyopathy developing several weeks and months after initiation of clozapine medication; however, this study revealed asymptomatic heart failure as a third cardiac adverse reaction of clozapine that develops years later. Therefore, regular monitoring of NT-proBNP contributes to improving long-term prognosis of treatment-resistant schizophrenia with prescribed clozapine.

## 1. Introduction

Clozapine is one of the most efficacious antipsychotics and the sole antipsychotic approved for treatment-resistant schizophrenia; however, its tolerability profile is complicated by a lowest incidence of extrapyramidal side effects and a high frequency of weight gain and metabolic complications, along with other difficult-to-manage adverse effects, such as liver damage, thermoregulatory disturbances, and hypersalivation [1,2,3,4]. Notably, among adverse reactions of clozapine, agranulocytosis is the most well-recognized life-threatening complication [1,5,6,7], whereas cardiotoxicity, such as myocarditis, cardiomyopathy, and myocardial infarction, as well as non-infectious pneumonia, pulmonary embolism, deep vein thrombosis, and convulsion, have been evaluated to carry a higher relative mortality compared to agranulocytosis [1,7,8,9,10]. Therefore, clozapine remains a double-edged therapeutic option due to these adverse reactions [1,2,3].

Notably, in East Asia, the proportion of individuals with low clozapine clearance (probably due to low CYP1A2 activity) is higher than Caucasians, raising concerns of clozapine-induced cardiotoxicity [11,12,13,14,15,16,17]. Most cases of clozapine-induced myocarditis develop within several weeks after clozapine initiation, whereas clozapine-induced cardiomyopathy typically manifests after several months of clozapine exposure [18,19,20]. Among clozapine-induced cardiotoxicities, the prevalence of clozapine-induced cardiomyopathy is approximately half that of clozapine-induced myocarditis, but case-fatality rates are comparable [1,18]. Therefore, the concern is that prevalence of clozapine-induced cardiomyopathy has been underestimated, since the development of clozapine-induced cardiomyopathy requires long-term clozapine exposure [1,2,21,22]. Indeed, reported prevalence of clozapine-induced cardiomyopathy was 1.4% in Oceania, where cardiac function monitoring is conducted intensively, versus 0.2% in other regions [18]. In other words, these regional differences in the prevalence of clozapine-induced cardiotoxicity should be viewed with concern for underestimation, especially in situations where intensive/continuous cardiac function monitoring is not being implemented. Despite concerns about the high risk of cardiotoxicity with clozapine in East Asia [11,17], the actual prevalence associated with clozapine-induced cardiotoxicity in East Asia, including Japan and China, is lacking, especially when only two Japanese case reports regarding clozapine-induced cardiomyopathy have been published to date [16,21]. So far, clozapine-induced cardiotoxicities have been considered to be type B adverse reactions of clozapine as rare, unpredictable, and not dose-dependent events [1,19,23,24,25]. However, the dose-dependent and time-dependent features suggest that the pathophysiology of clozapine-induced cardiotoxicities possibly do not necessarily comprise type B adverse reactions. Indeed, our recent preclinical studies demonstrated that clozapine inhibited protein phosphatase 2A (PP2A) activity together with enhancement of Src activity, possibly contributing to mechanisms of clinical efficacies and adverse reactions of clozapine [26,27]. Given the evidence that aberrant Src activation and reduced PP2A activity contribute to the exacerbation of pathophysiology in cardiomyopathy, myocardial infarction, and arrhythmogenesis [28,29,30,31,32,33,34], it cannot be excluded that clozapine has modest but clinically relevant pharmacodynamic cardiotoxic risks. In other words, clozapine-induced cardiotoxicity may not be attributable solely to a type B adverse reaction related to individual vulnerability, as it may also involve type A mechanisms associated with dose dependence, time dependence, and/or cumulative exposure, as well as potential interactions among these mechanisms [21,22,26,27,35]. Based on the fact that the majority of patients with treatment-resistant schizophrenia require decades of clozapine therapy [2,36,37], investigating not just clozapine-induced myocarditis/cardiomyopathy but also the broader impact of long-term clozapine exposure on cardiac function can provide critically important clinical information for improving long-term prognosis in this population.

Currently, heart failure (HF) has been defined as a global pandemic, with 64.3 million people estimated to suffer from heart failure worldwide in 2017 [38]. Although the prevalence of HFrEF (heart failure with reduced ejection fraction: EF < 40%) has been stable or even decreasing, the prevalence of HFpEF (heart failure with preserved ejection fraction: EF > 50%) has conversely been observed to be increasing, resulting in the increasing total prevalence of HF [38,39]. Notably, as opposed to HFrEF for which prognosis and survival have improved due to successful evidence-based therapies for HF, survival rates for HFpEF have yet to improve [38,39]. Prevalence of crude heart failure in China and Japan were reported to be lower than other regions, with 1.1% in China and 1.5% in Japan [38,40,41]; however, HFpEF accounts for 69% of patients with stage-C heart failure, defined as those currently experiencing symptomatic heart failure, and 51% of patients with stage-D heart failure, defined as those with persistent severe symptoms that markedly limit daily activities despite optimal medical and non-pharmacological therapies, suggesting that HFpEF has become the predominant heart failure phenotype and that its burden continues to increase in Japan [42]. As the burden of heart failure continues to grow, early initiation of guideline-directed medical therapy at stage-B heart failure, defined as structural or functional cardiac abnormalities in the absence of current symptoms, is increasingly recognized as contributing to the prevention of progression to stage-C heart failure and the reduction in long-term cardiovascular risk [39]. It is well-known that metabolic complication is a risk factor for HFpEF [43,44,45,46]. Considering the evidence that clozapine is a high-risk antipsychotic for metabolic complications [1,47,48,49,50], the hypothesis that long-term clozapine exposure contributes to the development of HF via metabolic complications is plausible. Based on these backgrounds, this study determined the prevalence of cardiac dysfunction among patients with long-term clozapine prescriptions, and explored the pathophysiology of cardiac dysfunction in patients prescribed clozapine.

## 2. Materials and Methods

This study was approved by the Clinical Research Ethics Review Committee of Mie University Hospital (approval no. H2022-200, 27 October 2022) and the Ethics Committee of the First Affiliated Hospital of China Medical University (approval no. 2024-1040, 20 November 2022). The requirement for obtaining individual informed consent was waived because the study used existing clinical data and posed minimal risk to the participants. The study information was publicly disclosed in accordance with the national guidelines of Japan and China, and patients were given the opportunity to opt out.

### 2.1. Cases

This study retrospectively reviewed the medical records of patients attending and admitted to two affiliated facilities of Mie University (Tsu, Japan) and three psychiatric-specialized institutions of the medical alliance in the Department of Psychiatry, the First Affiliated Hospital of China Medical University (Shenyang, Liaoning Province, China).

In Japan, clozapine was approved as the monotherapy for treatment-resistant schizophrenia in 2009 (clozapine is not approved for administration in combination with other antipsychotic medication). Information on concomitant non-antipsychotic medications in the Japanese cohort is summarized in Supplementary Table S1. In China, clozapine was approved for the treatment of schizophrenia in 1976 (gradually put into widespread clinical use starting in 1980). In China, the concomitant medication of clozapine with other antipsychotics has not been prohibited; however, in this study, only cases receiving clozapine monotherapy were included to match Japanese conditions [1,51]. In China, according to medical insurance regulations, non-psychiatric drugs are not allowed to be combined for hospitalized patients with schizophrenia who have no confirmed physical illnesses. Only two patients in the Chinese cohort were administered propranolol concomitantly (Supplementary Table S1). These medication characteristics reflect the differences in psychiatric medical practices between China and Japan.

In Japan and China, the study included cases for which clinical records were available at the initiation period of clozapine prescription, as well as laboratory examination data in 2025 (from 1 January 2025 to 31 December 2025) required by the Japanese Clozaril Patient Monitoring Service, including serum/plasma levels of creatinine (Cre), estimated glomerular filtration rate (eGFR), creatine kinase (CPK), glycosylated hemoglobin A1c (HbA1c), triglycerides (TG), HDL cholesterol (HDL), LDL cholesterol (LDL), neutrophil (Neut), lymphocyte (Lyph), monocyte (Mono), eosinophil (Eo), basophil (Baso), platelet (PLT), and cardiac function, such as brain natriuretic peptide (BNP), NT-proBNP, or left ventricular ejection fraction (LVEF). In the Japanese cohort, NT-proBNP and LVEF are regularly determined to detect cardiomyopathy and myocarditis at an early stage (asymptomatic stage). In the Chinese cohort, LVEF, BNP, or NT-proBNP monitoring is performed on asymptomatic patients only with their informed consent (for detection of cardiac dysfunction at an early stage or asymptomatic stage). In this study, LVEDV (left ventricular end-diastolic volume), LVESV (left ventricular end-systolic volume), IVST (interventricular septal thickness), PWT (posterior wall thickness), and LVEF (left ventricular ejection fraction) were monitored using echocardiography. All echocardiographic data were reviewed and verified by cardiologists during the LVEF calculation process, and diagnoses were made accordingly. In this study, LVEF was used as a representative value for assessing cardiac function using echocardiographic monitoring. Cases in which the duration of clozapine administration could not be defined were excluded. Particularly, some cases where clozapine administration was initiated at another facility and transferred to the participating research facility were excluded, due to the fact that the observation period was over 16 years in Japan and over 45 years in China, which is longer than the mandatory record-keeping period for general medical facilities. Especially in Japan, cases were included for which plasma levels of clozapine (CLZL), desmethyl-clozapine (DMCL), and clozapine-N-oxide (CNOL), as well as the aforementioned laboratory examination data, could be collected at multiple time points; therefore, these cases were adopted for fixed-effects analysis.

### 2.2. Statistical Analyses

As defined in the 2025 Guidelines for the Diagnosis and Treatment of Heart Failure (JCS/JHFS), patients with BNP > 35 pg/mL, NT-proBNP > 125 pg/mL, or LVEF < 50% were evaluated as having HF more severe than stage-B [39]. Prevalence of HF in Japan and China were calculated using real-world databases, the Japan Medical Data Center and the national urban employee basic medical insurance, respectively (crude HF prevalence) [40,41]. Age-standardized HF prevalence in Japan and China was derived from the crude HF prevalence using WHO’s World Standard Population model [52].

Random effects of daily dose and exposure duration of clozapine, Cre, eGFR, CPK, HbA1c, TG, HDL, LDL, neutrophils, lymphocytes, monocytes, eosinophils, basophils, and platelets on NT-proBNP and LVEF were analyzed by multiple regression analysis (MRA) using Gretl for Windows (v2025c), and analysis of covariance (ANCOVA) by SPSS for Windows (ver30.0, IBM, Armonk, NY, USA) was conducted.

For cases who underwent multiple NT-proBNP or LVEF measurements (in Japanese cases), fixed effects analyses were conducted using a linear mixed model for repeated measures, in which clozapine exposure duration was a linear covariate (LMM: lme4 ver2.0-1 in R) as temporal-dependent fixed effect. Additionally, fixed effects of daily dose and exposure duration of clozapine, as well as laboratory data (eGFR, CPK, HbA1c, tg, HDL, LDL), blood cell counts (neutrophils, lymphocytes, monocytes, eosinophils, basophils, and platelets), and plasma levels of clozapine and its metabolites (CLZ, DMC, CNO) on NT-proBNP and LVEF were determined using a hierarchical linear regression model with robust standard error (HLM: Gretl for windows, v2025c) [53,54,55].

## 3. Results

### 3.1. Prevalence of HF Associated with Clozapine

The total number of cases of clozapine prescribed alone without other concomitant antipsychotics, and for whom medical records were available in Japan (2009–2025) and China (1980–2025), were 99 (64 males and 35 females) and 216 (120 males and 96 females), respectively. In Japanese cases, NT-proBNP was measured in all 99 cases and LVEF was measured in 55 cases (39 males and 16 females). In Chinese cases, LVEF, NT-proBNP, and BNP were measured in 174 cases (97 males and 77 females), 82 cases (45 males and 37 females), and nine cases (3 males and 6 females), respectively (Table 1 and Table 2 and Supplementary Figure S1).

The number of patients with stage-B HF, defined by “JCS/JHFS 2025 Guideline on Diagnosis and Treatment of Heart Failure” (asymptomatic with NT-proBNP > 125 pg/mL, BNP > 35 pg/mL or LVEF < 50%), or more severe [39] equaled nine cases in Japan (crude prevalence = 9.1%, 95%CI: 4.2–16.6%) and 13 cases in China (crude prevalence = 6.0%, 95%CI: 3.2–10.1%) (Supplementary Table S2).

Age-standardized HF prevalence in general population of Japan and China were 1.9% and 0.53%, respectively. Age-standardized HF prevalence in patients with clozapine prescriptions in Japan and China were 7.2% and 2.9%, respectively. Therefore, the odds ratio for age-standardized HF prevalence among patients with prescribed clozapine in Japan and China were 3.2 (95%CI: 1.4–6.4) and 6.9 (95%CI: 3.6–12.0), respectively, compared to general population [40,41]. Neither regional nor sexual differences in the prevalence of HF associated with clozapine were detected in clozapine-prescribed patients. Therefore, the risk of stage-B HF associated with clozapine between Japan and China was almost equal.

Among nine Japanese cases, one case was diagnosed with cardiomyopathy/HFrEF associated with clozapine exposure (incidence = 1.0%, 95%CI: 0–5.5%) who had discontinued the clozapine prescription [21]. One case had renal dysfunction (incidence = 1.0%, 95%CI: 0–5.5%). Two cases had cardiomyopathy and myocardial infarction (incidence = 2.0%, 95%CI: 0.2–7.1%). Among 13 cases in China, there were no obvious cases with cardiomyopathy/HFrEF associated with clozapine exposure in China. Four cases had poorly controlled diabetes (incidence = 1.9%, 95%CI: 0.5–4.7%), but the other nine cases could not identify severe complications (incidence = 4.2%, 95%CI: 1.9–7.8%).

Concomitant non-antipsychotic medications in the cohorts of this study are summarized in Supplementary Table S1. In the Chinese cohort, two patients receiving propranolol in combination were included. Propranolol is metabolized by CYP1A2/2C19/2D6, but the cardiac function of patients receiving propranolol remained normal [56]. In the Japanese cohort, various concomitant non-antipsychotic medications were administered in clozapine-prescribed patients; however, there were no concomitant non-antipsychotic medications metabolized by CYP1A2, which is major metabolic enzyme for clozapine (Supplementary Tables S1 and S2).

### 3.2. Fixed-Effects Analyses in the Japanese Cohort

#### 3.2.1. Impacts of Clozapine Exposure Periods on NT-proBNP and LVEF in Japan Using LMM

**Figure 1:**
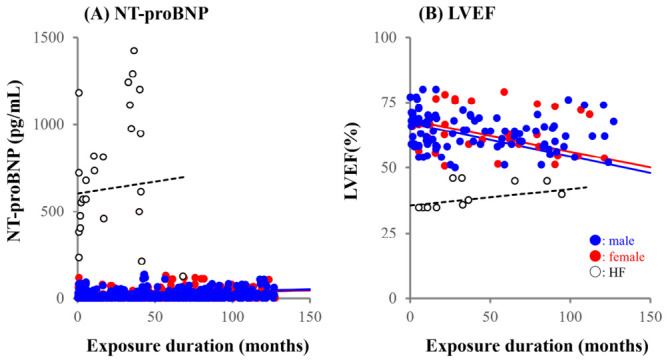
Impacts of clozapine exposure duration on NT-proBNP (**A**) and LVEF (**B**) detected by LMM in Japan. Ordinates indicate NT-proBNP level (pg/mL) and LVEF (%), and abscissas indicate duration of clozapine exposure (months). Open circles represent cases indicating abnormalities in NT-proBNP (>125 pg/mL) or LVEF (<50%). Blue and red circles represent males and females within reference ranges of NT-proBNP and LVEF. Solid and dotted lines indicate significant and non-significant trends, respectively, as detected by linear mixed model for repeated measures (LMM). Fixed effects of NT-proBNP in males and females by LMM were 0.17 ± 0.06 (β ± SE), t(463.8) = 2.96 (*p* < 0.01) and 0.16 ± 0.06, t(170.5) = 2.5 (*p* < 0.05), respectively. Fixed-effects LVEF of males and females by LMM were −0.11 ± 0.02, t(45.3) = −4.2 (*p* < 0.01) and −0.10 ± 0.04, t(32.4) = −2.3 (*p* < 0.05), respectively.

The database in Japan contained multiple time points of NT-proBNP, LVEF, and other laboratory data, allowing for fixed-effect analysis. Temporal impacts of clozapine exposure duration on NT-proBNP and LVEF were determined using LMM. Among all cases with and without cardiac functional abnormalities, significant relations between NT-proBNP/LVEF and clozapine exposure duration could not be detected (Figure 1). Among cases with HF (NT-proBNP > 125 pg/mL or LVEF < 50%) alone, relations between NT-proBNP/LVEF and clozapine exposure duration could also not be detected (Figure 1). However, among cases without HF (excluding cases with NT-proBNP > 125 or LVEF < 50), prolonged clozapine exposure duration related to increasing NT-proBNP levels and decreased LVEF (Figure 1). No sex-dependent differences were detected in the relations between clozapine exposure duration and NT-proBNP/LVEF. These results suggest the possibility that HF associated with clozapine has type B adverse reactions, but clozapine possibly has time-dependent cardiotoxicity, such as type A adverse reactions.

#### 3.2.2. Impacts of Exposure Periods and Daily Dose of Clozapine on NT-proBNP and LVEF in Japan Using HLM

The relations between daily dose and exposure duration of clozapine and NT-proBNP and LVEF in cases of prescribed clozapine without HF (excluding cases with NT-proBNP > 125 and LVEF < 50) were analyzed using fixed effects of HLM with robust standard error. Among males + females, males, and females, both exposure duration and daily dose of clozapine alone positively related to NT-proBNP and negatively to LVEF (Table 3). These results were identical to the results using LMM. When these independent variables were combined, among males + females, males, and females, clozapine exposure duration was positively related to NT-proBNP and negatively related to LVEF, but significant relations between daily clozapine dose and NT-proBNP/LVEF could not be detected (Table 3). Therefore, these results suggest that exposure duration of clozapine is probably predominant factor for cardiac function compared to clozapine daily dose.

#### 3.2.3. Impacts of Laboratory Data and Blood Cell Counts on NT-proBNP and LVEF in Japan Using HLM

The relations between laboratory examination data (eGFR, CPK, HbA1c, TG, HDL, and LDL) and blood cell counts (neutrophils, lymphocytes, monocytes, eosinophils, basophils, and platelets) and NT-proBNP and LVEF in cases of prescribed clozapine without HF (excluding cases with NT-proBNP > 125 and LVEF < 50) were also analyzed using fixed effects of HLM with robust standard error. From laboratory examination data required by the Clozaril Patient Monitoring Service, CPK, eGFR, and monocytes related to cardiac dysfunction (NT-proBNP and LVEF), whereas any other laboratory examination values did not significantly relate with NT-proBNP or LVEF (Table 4).

#### 3.2.4. Impacts of Plasma Clozapine Levels on NT-proBNP and LVEF in Japan Using HLM with Robust Standard Error

The relations between plasma levels of clozapine and its metabolites and NT-proBNP and LVEF in cases of prescribed clozapine without HF (excluding cases with NT-proBNP > 125 and LVEF < 50) were also analyzed using fixed effects of HLM with robust standard error. Plasma clozapine levels alone positively related to NT-proBNP but not to LVEF in males + females, males, and females (Table 5). Considering the pronounced interindividual variability in clozapine clearance within the Japanese population [11], these discrepancies suggest that such pharmacokinetic heterogeneity in Japan possibly plays a major role in individual vulnerability to increasing NT-proBNP or cardiac dysfunction associated with long-term clozapine medication.

### 3.3. Random-Effects Analyses in Both Japanese and Chinese Cohorts

#### 3.3.1. Impacts of Daily Dose and Exposure Duration of Clozapine, Monocytes, eGFR, and CPK on NT-proBNP and LVEF Using MRA

The data from China contained single points of NT-proBNP, LVEF, and other laboratory data in 2025 alone. Therefore, random-effect analyses were conducted using pooled data in both China and Japan in 2025. The effects of clozapine exposure duration, CPK, eGFR and monocytes, which were detected to have significant impacts on NT-proBNP and LVEF in Japanese cases by fixed-effects analyses, as well as clozapine daily dose, were determined using MRA. Among all the cases with and without abnormalities of NT-proBNP and LVEF, significant relations between any independent variables (clozapine exposure duration, clozapine daily dose, eGFR, CPK, and monocytes) to NT-proBNP could not be detected. In contrast, among groups without abnormalities (excluding cases with NT-proBNP > 125, BNP > 35, and LVEF < 50), NT-proBNP levels were positively related to clozapine exposure duration and monocytes in Japanese cases (males + females). In Chinese cases (males + females), NT-proBNP levels were positively related to clozapine exposure duration alone. Among all males (Japanese + Chinese) and females (Japanese + Chinese), NT-proBNP did not relate to any independent variables (Table 6). Significant relations between LVEF and five independent variables could not be detected (Table 6).

#### 3.3.2. Impacts of Exposure Duration of Clozapine and Monocytes on NT-proBNP Using ANCOVA

**Figure 2:**
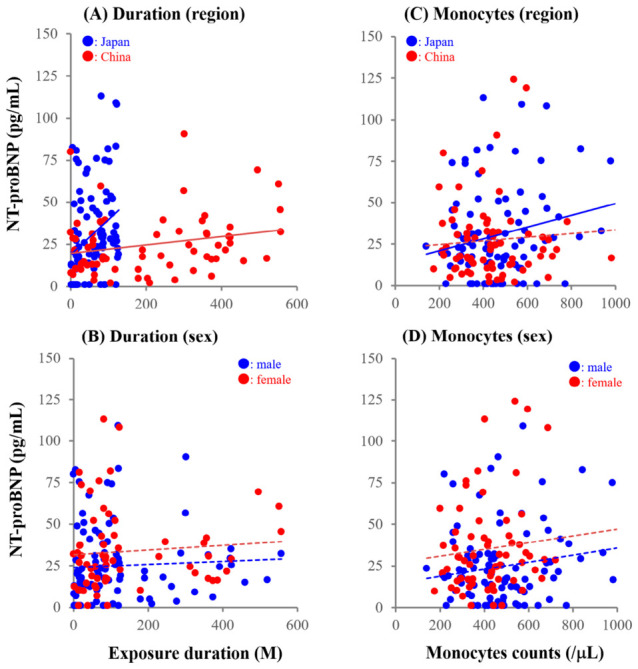
Impacts of clozapine exposure duration and monocytes on NT-proBNP. Panels (**A**,**B**) indicate relations between clozapine exposure duration (months) and NT-proBNP levels (pg/mL), disaggregated by region (Japan vs. China) and sex (males vs. females). Panels (**C**,**D**) indicate relations between monocytes counts (/μL) and NT-proBNP levels (pg/mL), disaggregated by region (Japan vs. China) and sex (males vs. females). Blue and red circles in panels (**A**,**C**) indicate Japanese and Chinese cases, respectively. Blue and red circles in panels (**B**,**D**) indicate males and females, respectively. Solid and dotted lines indicate significant and non-significant trends detected by ANCOVA. In panel (**A**): F_region_(1,160) = 0.1 (*p* > 0.1), F_duration_(1,160) = 13.6 (*p* < 0.05), and F_region*duration_(1,160) = 9.3 (*p* < 0.05). In panel (**B**): F_sex_(1,160) = 1.9 (*p* > 0.1), F_duration_(1,160) = 0.3 (*p* > 0.1), and F_sex*duration_(1,160) = 0.1 (*p* > 0.1). In panel (**C**): F_region_(1,166) = 0.8 (*p* > 0.1), F_monocyte_(1,166) = 6.9 (*p* < 0.01), and F_region*monocyte_(1,166) = 1.9 (*p* > 0.1). In panel (**D**): F_sex_(1,295) = 0.1 (*p* > 0.1), F_monocyte_(1,295) = 5.5 (*p* < 0.05), and F_sex*duration_(1,295) = 0.5 (*p* > 0.1).

Interestingly, in above section, the significant relations between NT-proBNP and clozapine exposure duration could not be detected when China and Japan were pooled. These discrepancies may be due to confounding factors related to clinical different conditions between Japan and China, such as clozapine exposure duration (Table 1 and Table 2). Therefore, impacts of clozapine exposure duration and monocytes on NT-proBNP were determined using ANCOVA. Positive impacts of clozapine exposure duration on NT-proBNP in Japan (males + females) were greater than in China (males + females) (Figure 2). In an analysis including only cases with exposure duration less than 100 months, the difference between Japan and China in the positive trends of clozapine exposure duration on NT-proBNP could not be detected (Supplementary Figure S2). Significant impacts of exposure duration on NT-proBNP in males (Japanese + Chinese) and females (Japanese + Chinese) were not detected (Figure 2). In contrast, positive impacts of monocytes on NT-proBNP in Japan were detected, but these could not be detected in China (Figure 2). Both monocytes of males and females (Japanese + Chinese) were not related to NT-proBNP (Figure 2).

### 3.4. Impacts of Daily Dose and Exposure Duration of Clozapine on Monocytes

#### 3.4.1. Fixed Effects of Exposure Duration and Daily Dose of Clozapine on Monocytes

**Figure 3:**
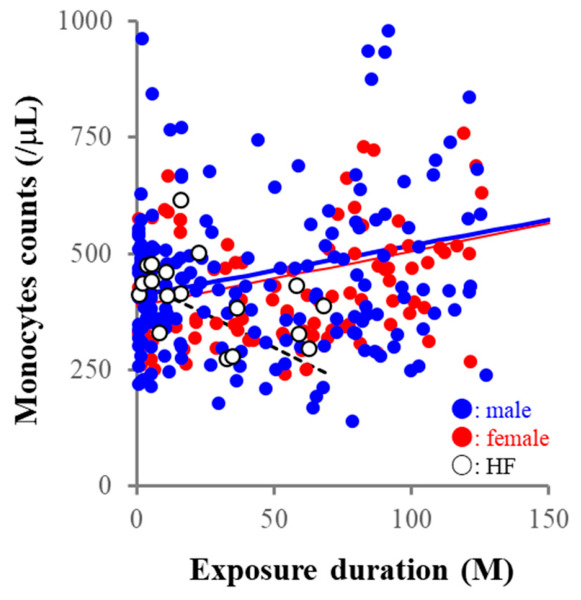
Impacts of exposure duration on monocytes using LMM. Panel indicates fixed effects of clozapine exposure duration (months) on monocytes counts (/μL) in Japanese cases by LMM. Blue and red circles indicate males and females, respectively. Open circles represent cases with abnormalities in NT-proBNP (>125 pg/mL) or LVEF (<50%). Solid and dotted lines indicate significant and non-significant in LMM. Fixed effects of exposure duration on monocytes in males and females by LMM were 0.91 ± 0.41 (β ± SE), t(102.1) = 2.22 (*p* < 0.05) and 1.01 ± 0.50, t(47.3) = 2.0 (*p* < 0.05), respectively.

The results in the above sections suggest that clozapine exposure duration may increase NT-proBNP levels in cases of prescribed clozapine by increasing monocytes. Within the Japanese cohort without HF (males + females, males and females), LMM detected the positive fixed effects of exposure duration of clozapine on monocytes counts (Figure 3).

Furthermore, HLM with robust standard error also detected the positive fixed effects of plasma levels and exposure duration of clozapine on monocytes counts, but daily dose of clozapine did not relate to monocytes (Table 7).

#### 3.4.2. Random Effects of Exposure Duration and Daily Dose of Clozapine on Monocytes

**Figure 4:**
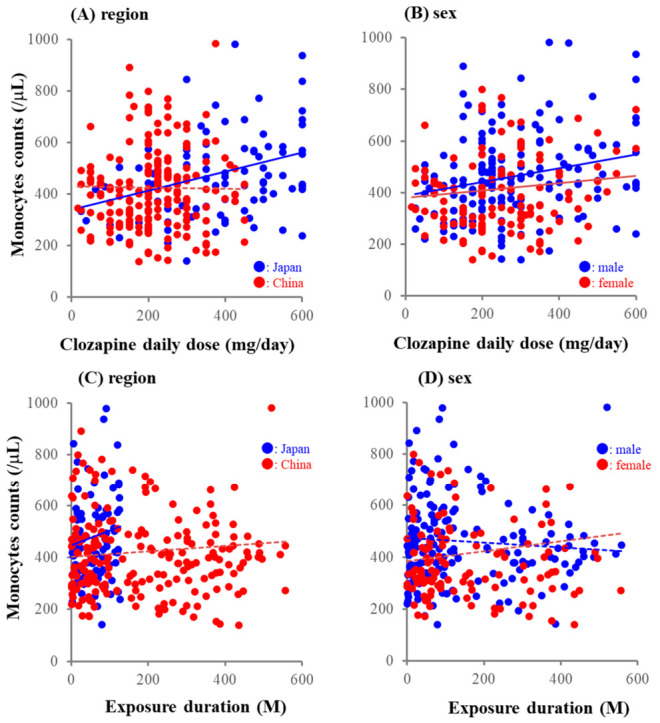
Impacts of daily dose and exposure duration of clozapine on monocytes using ANCOVA. Panels (**A**,**B**) indicate impacts of clozapine daily dose (mg/day) on monocytes counts (/μL), disaggregated by region (Japanese vs. Chinese cases) and sex (males vs. females). Blue and red circles indicate Japanese and Chinese cases in panel (**A**) and males and females in panel (**C**). In panel (**A**): F_region_(1,295) = 1.9 (*p* > 0.1), F_dose_(1,295) = 2.7 (*p* > 0.05), and F_region*dose_(1,296) = 4.0 (*p* < 0.05). In panel (**B**): F_sex_(1,295) = 0.1 (*p* > 0.1), F_dose_(1,295) = 5.5 (*p* < 0.05), and F_sex*dose_(1,295) = 0.5 (*p* > 0.1). In panel (**C**): F_region_(1,287) = 0.6 (*p* > 0.1), F_duration_(1,287) = 1.4 (*p* > 0.1), and F_region*duration_ (1,287) = 2.0 (*p* > 0.1). In panel (**D**): F_sex_(1,287) = 3.2 (*p* > 0.05), F_duration_(1,287) = 3.1 (*p* > 0.05), and F_sex*duration_(1,287) = 0.3 (*p* > 0.1).

In contrast to fixed effects in Japanese cases, among cases combined Japanese and Chinese cohorts without HF, positive random effects of clozapine daily dose on monocytes were detected in Japan (males + females), males (Japanese + Chinese) and females (Japanese + Chinese), but not in China (males + females) by ANCOVA (Figure 4).

Positive random effect of clozapine exposure duration on monocytes were detected in Japan (males + females), but not in males (Japanese + Chinese), China (males + females), males (Japanese + Chinese) or females (Japanese + Chinese) by ANCOVA (Figure 4).

Similar to the results from ANCOVA, positive random effects of clozapine daily dose on monocytes were detected in all Japanese (males + females), as well as males (Japanese + Chinese) and females (Japanese + Chinese), but not for clozapine exposure duration by MRA (Table 8).

## 4. Discussion

This study revealed several important findings regarding the impacts of long-term clozapine administration on cardiac function in East Asian populations (including Japan and China). This study suggests the possibility that cardiotoxicities associated with clozapine exposure may not only comprise well-known acute myocarditis, occurring within weeks, and subacute cardiomyopathy, developing over several months, but also a third toxicity, in which cardiac dysfunction gradually progresses over several years (probably within one hundred months), ultimately manifesting to suggest HFpEF. Fixed- and random-effects analyses detected several risk factors for heart failure associated with clozapine, including prolonged exposure duration, higher plasma level of clozapine, and increasing monocytes. Importantly, over 70% of cases with heart failure associated with clozapine identified in this study did not have metabolic complications. Considering the facts that clozapine remains the sole effective antipsychotic for patients with treatment-resistant schizophrenia, patients with treatment-resistant schizophrenia must require long-term clozapine medication as their sole therapeutic option [1,2]. Therefore, our findings underscore the clinical importance for psychiatrists to maintain ongoing surveillance of cardiac function in patients who have already been receiving clozapine for extended periods to improve long-term prognosis of treatment-resistant schizophrenia with prescribed clozapine.

### 4.1. Clozapine and Cardiac Function

Clozapine-induced cardiotoxicity has a time-dependent characteristic, as the risk of clozapine-induced myocarditis is high for several months, with a subsequently increasing risk for clozapine-induced cardiomyopathy after initiation of clozapine intake [18,23,57,58]. Based on these clinical findings, LVEF monitoring using echocardiography has been considered important and useful diagnostic marker for cardiac dysfunction in cases of long-term clozapine exposure [18,59]. Indeed, although globally unified consensus regarding the diagnostic criteria for clozapine-induced cardiomyopathy has yet to be established, LVEF < 50% and decreasing LVEF of over 10% from baseline have nevertheless been adopted as diagnostic thresholds in various clinical studies [18,59]. However, this study elucidated that in cases with long-term clozapine medication, the majority of HF are suggestive of HFpEF, which is insensitive to detection with LVEF monitoring or asymptomatic HF. Therefore, compared to LVEF monitoring using echocardiography, NT-proBNP is considered a more sensitive and clinically useful biomarker for stage-B HF associated with clozapine.

Monitoring LVEF using echocardiography requires costly equipment and skilled sonographers and cardiologists to obtain reliable LVEF measurements. The technical and financial burden to monitoring LVEF can be substantial, since prescriptions of clozapine are typically only permitted for psychiatrists who are board-certified psychiatrists registered with the Clozaril Patient Monitoring Service in specialized psychiatric hospitals. In contrast, NT-proBNP is a simple blood test that avoids the various logistical burdens associated with echocardiography, making it a more feasible clinical tool for routine HF monitoring in psychiatric settings. Moreover, cases with asymptomatic HF or are suggestive of HFpEF identified in this study were partially complicated with poorly controlled diabetes, asymptomatic old myocardial infarction, and chronic renal dysfunction, which are well-known complications for clozapine medication. These facts further support the notion that NT-proBNP may serve as a valuable biomarker for comprehensive screening of cardiac dysfunction associated with long-term clozapine therapy. Therefore, the present findings emphasized the potential utility of NT-proBNP as a practical and sensitive indicator for detecting early cardiac impairment in patients receiving prolonged clozapine treatment.

The distinct pathophysiological bases between HFrEF and HFpEF are involved in their divergent preceding complications/comorbidities and subsequent inflammatory pathways [60,61,62,63]. HFrEF is typically preceded by the acute/chronic loss of cardiomyocytes induced typically by ischemia, genetic mutations, or myocarditis, whereas HFpEF is characteristically preceded by clusters of non-cardiac metabolic comorbidities, such as obesity, type 2 diabetes, and renal dysfunction [60,61,62,63]. These different triggers dictate the nature of the inflammatory response; in HFrEF, systemic and cardiac inflammation occur as a secondary reaction to cardiomyocyte damage and necrosis. In contrast, HFpEF posits that metabolic risk factors drive chronic, low-grade systemic inflammation, which acts as a primary mediator of early microvascular endothelial dysfunction and subsequent myocardial stiffening [60,61,62,63,64]. Considering the evidence that clozapine is a high-risk antipsychotic for metabolic complications [1,47,48,49,50], the hypothesis that long-term clozapine exposure contributes to the development of HFpEF via metabolic complications is easily plausible. However, it should be considered that causes of stage-B HF associated with clozapine possibly comprise not just clozapine-induced metabolic complications but also other pathophysiology, as clozapine itself possesses pharmacological targets that may directly or indirectly affect the pathophysiology of HFpEF since over 70% of the cases with asymptomatic HF identified in this study did not have metabolic complications.

In this study, positive relations in patients with long-term clozapine medication were observed among clozapine (exposure duration, plasma levels, and daily dose), NT-proBNP, and monocytes. Early inflammatory reactions following clozapine initiation frequently involve eosinophilic responses [65]. A recent multicenter cohort study demonstrated that eosinophilia occurred in approximately half of the patients who developed inflammatory adverse events during the titration phase, typically appearing shortly after the onset of fever or C-reactive protein elevation [65]. Hyperactivation of eosinophilic responses is also considered to play an important role in the pathophysiology of clozapine-induced myocarditis [57,66]. This temporal pattern suggests that eosinophilia may represent a component of the broader inflammatory cascade triggered by clozapine rather than an isolated hematologic abnormality [57,66]. Growing evidence suggests that clozapine-induced inflammatory reactions also involve coordinated activation of monocytic responses [67,68]. However, to our knowledge, both monocyte and eosinophil responses have been limited to observations within the first few weeks of clozapine administration, but there are no reports targeting the slow monocyte response observed several years or more after clozapine exposure, as identified in this study. Both clinical and preclinical studies reported that increasing counts and responses of monocytes are potentially involved in decreasing cardiac function in patients and animal models of HFpEF, suggesting that by maintaining and promoting chronic inflammation associated with monocytes, they play an important role in the pathomechanism of HFpEF as a driver of diastolic dysfunction [69,70,71]. A pharmacodynamic preclinical study reported that clozapine activated Src and decreased PP2A activities [26,27]. Activation of Src activity alone can force cells to differentiate into the monocytic lineage and attenuate pathway to neutrophils [72,73,74,75]. Additionally, decreased PP2A activity in monocytes plays important roles in foam cell formation and initiation of atherosclerosis via the p38/CD36 signaling complex [76]. Considering these preclinical and clinical findings, the results in this study demonstrate that the positive relations between monocytes and NT-proBNP could be, at least partially, involved in the pathophysiology of stage-B HF associated with clozapine as a potentially novel pathophysiology.

CYP enzyme activity plays a critical role in determining clozapine-induced cardiotoxicity risk. CYP1A2, the primary enzyme responsible for clozapine clearance via N-demethylation, reduces cardiotoxicity risk when active; reduced CYP1A2 activity—due to smoking cessation, CYP1A2-inhibiting comedications, or Asian ancestry—elevates plasma clozapine concentrations and increases myocarditis risk [77,78]. Conversely, CYP3A4 predominantly catalyzes clozapine N-oxidation, generating reactive oxygen species and nitrenium intermediates implicated in direct cardiotoxic bioactivation, particularly within cardiac mitochondria [79,80]. CYP2D6 contributes modestly to clozapine clearance; poor metabolizers may experience elevated plasma levels, yet CYP2D6 also catalyzes nitrenium formation, making its net cardiotoxic contribution bidirectional [79,81]. Therapeutic drug monitoring guided by individual CYP phenotype therefore represents a rational strategy for minimizing clozapine cardiotoxicity [78]. In this study, only two patients in the Chinese cohort were prescribed propranolol, which is metabolized by CYP1A2, CYP2C19, and CYP2D6 [77,78], as concomitant medications, but their cardiac function remained within normal limits. In contrast, in the Japanese cohort, a number of concomitant medications, the majority of which are metabolized by CYP3A4 but not by CYP1A2, possibly contributed in suppressing rather than increasing cardiotoxicity [79,80].

Based on these pharmacokinetic findings, application of internationally clozapine titration protocol to Japanese population has recently been concerned and considered to be risk for myocarditis associated with clozapine exposure, since clozapine clearance in East Asians is lower than other regions [11,17,82]. Indeed, in Japan, the initial titration protocol is strictly regulated by the Pharmaceuticals and Medical Devices Agency (PMDA). According to the official prescribing information, clozapine is initiated at 12.5 mg on day 1, followed by 25 mg once daily on day 2. From day 3 onward, the dose may be increased by 25 mg per day, depending on clinical response, with the general recommendation to titrate up to 200 mg/day over approximately three weeks. When the daily dose exceeds 50 mg, it should be administered in two or three divided doses. The recommended maintenance dose ranges from 200 to 400 mg/day, given in two or three divided doses, with adjustments made according to symptom severity and tolerability. Importantly, each dose increase must be separated by at least four days, and the increment should not exceed 100 mg/day. The maximum permitted daily dose under Japanese regulations is 600 mg/day. In contrast, although clozapine prescriptions in China allow greater clinical discretion for the treating psychiatrist, the titration schedule itself is generally faster than that mandated in Japan. Indeed, the standard initial titration protocol in China is that clozapine is initiated at 12.5–25 mg daily, with gradual escalation to 100–300 mg daily within 1–2 weeks, up to a maximum of 600 mg daily. This indicates that Chinese patients typically reach 100–300 mg/day within the first 1–2 weeks, which represents a more rapid escalation compared with the strictly regulated Japanese PMDA protocol, which recommends titration to 200 mg/day over approximately three weeks. Therefore, titration method in the initial stages of clozapine administration may not be involved in the pathomechanisms of stage-B HF associated with clozapine. Instead, impacts of plasma levels and exposure duration of clozapine plays important roles in the pathomechanisms of stage-B HF associated with clozapine. In spite of shorter exposure duration in Japan than in China, there was no difference in the prevalence of HF between the two countries. This is probably explainable by the higher clozapine daily dose in Japan compared to China, and the results that the impacts of exposure duration on HF may be more pronounced in exposure periods within 100 months, as suggested by the results of this study. Furthermore, inter-individual variability in CYP1A2 and CYP2D6 activity, partly determined by genetic polymorphisms, may modulate cardiotoxicity severity by altering plasma clozapine levels. Although direct evidence linking these polymorphisms to cardiac outcomes remains limited, reduced CYP1A2 activity variants (*1C/*1D) have been associated with elevated clozapine concentrations and metabolic complications, suggesting that pharmacogenetic profiling may help identify patients at higher risk of clozapine associated cardiac dysfunction [79,81,83,84].

This study succeeded in demonstrating the high risk of asymptomatic HF, including HF suggestive of HFpEF, as a novel potential concern in long-term clozapine administration. However, there are several limitations in this study that warrant mentioning. This study was observational in a small cohort and it cannot be ignored as that it may be probably subject to ecological fallacy. The prevalence of cardiomyopathy and HF associated with clozapine are subject to confounding, as information on potential confounder(s) may not be available and associations at the population level do not necessarily represent associations at the individual level (ecological fallacy). Indeed, the potential pathophysiology of stage-B HF associated with clozapine had a greater impact by fixed-effects analyses compared to random-effects analyses. Data from Chinese cases was limited to one single period in 2025. Further data collection over multiple periods in Chinese cases, considering the impacts of various confounding factors between Japan and China, could lead to a more detailed and accurate understanding regarding HF associated with long-term clozapine medication. Among asymptomatic HF cases, few cases (less than 50%) measured both NT-proBNP and LVEF. Clarifying whether HF associated with clozapine manifests predominantly as suggestive of HFpEF or HFrEF could possibly contribute to the development of a more rigorous and clinically meaningful pathophysiological framework. This study successfully revealed the possibility that long-term clozapine monotherapy is a potential risk factor for heart failure. However, identifying the relationship between clozapine’s clinical efficacy and its risk of cardiotoxicity is both clinically and pathophysiologically important; therefore, further studies are needed.

### 4.2. Limitations

This study successfully identified stage-B or more severe heart failure risk and its associated biomarkers in patients prescribed clozapine monotherapy in Japan (15 years) and China (45 years). The comparison between Japanese and Chinese cohorts itself contributed to clinically valuable findings in psychiatric medicine; however, this study has several limitations. While this study attempted to compare heart failure risk between the general population and patients with long-term clozapine medication, the influence of confounding factors could not be fully excluded. Patients with treatment-resistant schizophrenia may differ substantially from the general population in numerous respects, including smoking habits, obesity, hypertension, physical activity levels, and access to medical care. Further studies comparing clozapine-treated patients with clozapine-naive schizophrenic patients can improve the precision of heart failure risk estimates attributable to clozapine itself. Furthermore, a comparison of clozapine discontinuation rates and their underlying causes between the Japanese (15 year) and Chinese (45 year) cohorts may provide additional clinically important insights in psychiatric medicine.

## 5. Conclusions

This study revealed that the risk of asymptomatic HF in cases of long-term prescribed clozapine (57.5 ± 4.0 months in Japan and 208.1 ± 11.0 months in China) had approximately 3- and 6-folds higher odds in both Japan and China, respectively. Asymptomatic HF due to long-term clozapine exposure was both time dependent and concentration dependent, and the fixed effect of clozapine was more sensitive than the random effect. Therefore, it is highly likely that asymptomatic HF can be adequately predicted in routine clinical practice through regular NT-proBNP monitoring. Given that patients on long-term prescribed clozapine are resistant to other conventional antipsychotics and have few alternative treatment options, psychiatrists can contribute to improving the long-term prognosis of patients with treatment-resistant schizophrenia by adding regular NT-proBNP monitoring to conventional psychiatric symptom assessment.

## Figures and Tables

**Table 1:** Discriminative statistics in cases of prescribed clozapine in Japan.

		Japan								
		Male + Female			Male			Female		
	Reference Range	Mean	SE	N	Mean	SE	N	Mean	SE	N
Age (Y)		45.3	1.1	99	43.8	1.4	64	48.2	1.7	35
Dose (mg/day)		379.5	15.2	99	381.1	19.5	64	376.8	24.6	35
Administration duration (M)		57.5	4.0	99	53.0	5.2	64	65.7	6.2	35
NT-proBNP	<125 pg/mL	60.7	17.7	99	71.3	27.2	64	41.3	5.4	35
BNP	<35 pg/mL	-	-	-	-	-	-	-	-	-
LVEF	<55%	59.2	1.1	55	58.4	1.3	39	61.1	2.1	16
eGFR	>90 mL/min/1.73 m^2^	81.5	1.8	99	83.6	2.2	64	77.7	3.0	35
CPK	20–200 U/L	88.5	8.4	99	97.7	10.7	64	71.8	13.2	35
HbA1c	<6.5%	5.4	0.0	95	5.4	0.0	61	5.4	0.1	34
TG	<150 mg/dL	145.1	9.4	99	158.3	12.1	64	120.9	14.2	35
HDL	>40 mg/dL	55.2	1.6	95	50.4	1.7	63	64.5	2.9	32
LDL	<130 mg/dL	92.6	2.9	99	93.6	3.6	64	90.7	5.1	35
Neut	2500–8000/μL	4658.2	178.8	99	4560.4	220.7	64	4837.0	306.9	35
Lyph	1000–4000/μL	1711.8	67.4	99	1714.9	86.1	64	1706.1	109.1	35
Mono	100–700/μL	477.5	18.1	99	485.6	23.4	64	462.6	28.6	35
Eo	50–500/μL	213.9	28.9	99	240.2	40.9	64	165.7	31.7	35
Baso	25–100/μL	36.6	2.6	99	39.5	3.6	64	31.4	3.1	35
PLT	15–45 × 10^4^/µL	24.3	0.7	99	23.6	0.7	64	25.5	1.3	35
CLZL	350–600 ng/mL	607.7	37.0	98	573.7	45.8	63	668.8	62.4	35
DMCL	100–300 ng/mL	631.0	44.4	98	581.1	53.8	63	720.9	76.6	35
CNOL	(ng/mL)	160.0	12.7	98	147.8	11.6	63	182.0	28.8	35

Brain natriuretic peptide (BNP), left ventricular ejection fraction (LVEF), creatinine (Cre), estimated glomerular filtration rate (eGFR), creatine kinase (CPK), glycosylated hemoglobin A1c (HbA1c), triglycerides (TG), HDL cholesterol (HDL), LDL cholesterol (LDL), neutrophil (Neut), lymphocyte (Lyph), monocyte (Mono), eosinophil (Eo), basophil (Baso), platelet (PLT), plasma levels of clozapine (CLZL), desmethyl-clozapine (DMCL), clozapine-N-oxide (CNOL), standard error (SE).

**Table 2:** Discriminative statistics in cases prescribed clozapine in China.

		China								
		Male + Female			Male			Female		
	Reference Range	Mean	SE	N	Mean	SE	N	Mean	SE	N
Age (Y)		52.1	0.8	216	50.8	1.1	120	53.6	1.1	96
Dose (mg/day)		192.2	10.9	207	193.8	14.9	116	190.1	15.9	91
Administration duration (M)		209.7	6.7	215	218.3	8.9	120	198.9	10.2	95
NT-proBNP	<125 pg/mL	64.9	22.8	82	83.9	41.2	45	41.7	6.3	37
BNP	<35 pg/mL	84.9	36.1	9	29.9	1.4	3	112.4	51.7	6
LVEF	<55%	64.3	0.3	174	63.8	0.4	97	64.8	0.5	77
eGFR	>90 mL/min/1.73 m^2^	82.5	1.2	216	86.1	1.7	120	77.9	1.5	96
CPK	20–200 U/L	69.1	3.3	206	82.1	5.4	110	54.1	3.0	96
HbA1c	<6.5%	6.1	0.1	206	6.2	0.1	119	6.1	0.1	87
TG	<150 mg/dL	154.4	5.6	216	156.8	8.2	120	151.3	7.4	96
HDL	>40 mg/dL	43.4	0.9	216	41.3	1.2	120	46.1	1.2	96
LDL	<130 mg/dL	89.5	1.7	216	86.3	2.3	120	93.5	2.6	96
Neut	2500–8000/μL	3977.7	102.0	216	3934.0	120.3	120	4032.4	173.8	96
Lyph	1000–4000/μL	1991.4	43.0	216	2125.2	58.6	120	1824.2	59.1	96
Mono	100–700/μL	418.8	14.4	216	440.4	13.7	120	391.7	27.4	96
Eo	50–500/μL	158.7	9.9	216	186.7	14.8	120	123.6	11.6	96
Baso	25–100/μL	24.6	1.1	216	25.7	1.7	120	23.2	1.5	96
PLT	15–45 × 10^4^/µL	21.8	0.5	216	20.3	0.8	120	23.8	0.6	96

**Table 3:** Fixed effects of exposure duration and daily dose of clozapine on NT-proBNP and LVEF by HLM with robust standard error.

NT-proBNP								
Sex	F	*p*	Factor	β	SE	T	*p*	
Male + female	77.026	0.000	Duration	0.724	0.082	8.78	0.000	**
Male	38.461	0.000	Duration	0.598	0.096	6.2	0.000	**
Female	43.122	0.000	Duration	0.93	0.142	6.57	0.000	**
Male + female	9.517	0.003	Dose	0.045	0.015	3.09	0.000	**
Male	3.966	0.058	Dose	0.024	0.012	1.98	0.000	**
Female	6.757	0.014	Dose	0.122	0.047	2.6	0.000	**
Male + female	40.89	0.000	Duration	0.69	0.082	8.43	0.000	**
			Dose	0.02	0.013	1.55	0.124	
Male	20.225	0.000	Duration	0.59	0.103	5.73	0.000	**
			Dose	0.004	0.013	0.34	0.735	
Female	27.816	0.035	Duration	0.828	0.133	6.24	0.025	*
			Dose	0.083	0.029	2.89	0.102	
**LVEF**								
Sex	F	*p*	Factor	β	SE	T	*p*	
Male + female	33.216	0.000	Duration	−0.285	0.049	−5.76	0.000	**
Male	32.426	0.000	Duration	−0.323	0.057	−5.69	0.000	**
Female	84.323	0.000	Duration	−0.161	0.018	−9.18	0.000	**
Male + female	11.637	0.001	Dose	−0.021	0.006	−3.41	0.000	**
male	10.758	0.002	Dose	−0.021	0.006	−3.28	0.000	**
Female	12.661	0.003	Dose	−0.024	0.007	−3.56	0.000	**
Male + female	18.792	0.000	Duration	−0.255	0.052	−4.88	0.000	**
			Dose	−0.011	0.007	−1.57	0.122	
Male	16.994	0.000	Duration	−0.295	0.066	−4.49	0.000	**
			Dose	−0.008	0.009	−0.95	0.350	
Female	3.595	0.028	Duration	−0.167	0.025	−6.69	0.022	*
			Dose	−0.025	0.021	−1.17	0.274	

** *p* < 0.01, * *p* < 0.05.

**Table 4:** Fixed effects of laboratory data and blood cell counts on NT-proBNP and LVEF by HLM with robust standard error.

NT-proBNP									LVEF								
Sex	F	*p*	Factor	β	SE	T	*p*		Sex	F	*p*	Factor	β	SE	T	*p*	
Male + female	3.495	0.004	eGFR	−0.186	0.12	−1.55	0.261		Male + female	4.473	0.001	eGFR	0.28	0.085	3.29	0.002	**
			CPK	0.082	0.01	8.4	0.014	*				CPK	0.024	0.022	1.13	0.265	
			HbA1c	−21.33	8.695	−2.45	0.134					HbA1c	5.451	3.993	1.37	0.178	
			TG	−0.01	0.012	−0.77	0.521					tg	−0.008	0.008	−0.98	0.332	
			HDL	0.283	0.132	2.15	0.165					HDL	−0.050	0.078	−0.65	0.521	
			LDL	−0.276	0.096	−2.89	0.102					LDL	0.027	0.069	0.39	0.7	
Male	1.476	0.201	eGFR	−0.121	0.148	−0.82	0.416		Male	5.385	0.001	eGFR	0.255	0.099	2.57	0.015	*
			CPK	0.013	0.034	0.39	0.698					CPK	0.038	0.023	1.66	0.107	
			HbA1c	−19.277	10.218	−1.89	0.064					HbA1c	8.053	4.606	1.75	0.089	
			tg	0.014	0.033	0.43	0.668					tg	−0.001	0.009	−1.12	0.269	
			HDL	0.213	0.183	1.17	0.248					HDL	−0.125	0.1	−1.26	0.217	
			LDL	−0.042	0.065	−0.65	0.517					LDL	−0.076	0.073	−1.04	0.307	
Female	7.23	0.001	eGFR	−0.278	0.373	−0.75	0.461		Female	20.33	0.001	eGFR	0.208	0.054	3.83	0.019	*
			CPK	0.18	0.069	2.59	0.014	*				CPK	−0.352	0.056	−6.25	0.003	**
			HbA1c	−19.732	23.488	−0.84	0.407					HbA1c	6.474	3.927	1.65	0.175	
			tg	0.087	0.040	−2.16	0.039	*				tg	−0.036	0.027	−1.3	0.264	
			HDL	0.697	0.477	1.46	0.154					HDL	0.003	0.046	0.05	0.96	
			LDL	−0.689	0.445	−1.55	0.132					LDL	0.251	0.029	8.6	0.001	
Male + female	2.696	0.018	Neut	−0.002	0.001	−1.63	0.106		Male + female	2.485	0.034	Neut	0.001	0.001	1.05	0.3	
			Lyph	−0.003	0.005	−0.57	0.568					Lyph	0.003	0.002	1.77	0.083	
			Mono	0.048	0.017	2.82	0.006	**				Mono	−0.022	0.009	−2.53	0.014	*
			Eo	−0.011	0.016	−0.69	0.491					Eo	−0.003	0.011	−0.32	0.750	
			Baso	0.142	0.088	1.62	0.108					Baso	−0.001	0.045	−0.03	0.976	
			PLT	−0.301	0.386	−0.78	0.437					PLT	−0.041	0.174	−0.24	0.815	
Male	4.545	0.001	Neut	−0.002	0.001	−1.79	0.074		Male	5.000	0.001	Neut	0.001	0	1.06	0.399	
			Lyph	−0.002	0.003	−0.75	0.455					Lyph	0.002	0.001	1.5	0.273	
			Mono	0.049	0.018	2.72	0.007					Mono	−0.017	0.003	−5.72	0.029	*
			Eo	−0.016	0.009	−1.84	0.066					Eo	0.005	0.007	0.68	0.566	
			Baso	0.051	0.078	0.66	0.511					Baso	0.051	0.053	0.96	0.437	
			PLT	0.438	0.285	1.54	0.124					PLT	0.011	0.142	0.08	0.946	
Female	4.684	0.018	Neut	0.001	0.001	0.92	0.455		Female	3.021	0.036	Neut	0.004	0.002	2.16	0.084	
			Lyph	−0.004	0.004	−0.99	0.428					Lyph	0.009	0.006	1.63	0.164	
			Mono	0.04	0.012	3.19	0.001	**				Mono	−0.065	0.023	−2.8	0.038	*
			Eo	0.014	0.009	1.51	0.270					Eo	−0.032	0.023	−1.4	0.219	
			Baso	0.424	0.185	2.29	0.150					Baso	0.121	0.128	0.95	0.386	
			PLT	−1.627	0.66	−2.46	0.133					PLT	−0.371	0.403	−0.92	0.399	

** *p* < 0.01, * *p* < 0.05.

**Table 5:** Impacts of plasma clozapine levels on NT-proBNP and LVEF in Japan using HLM.

NT-proBNP								
Sex	F	*p*	Factor	β	SE	T	*p*	
Male + female	9.314	0.000	CLZ	0.0221	0.0095	2.33	0.022	*
			DMC	0.0077	0.0097	0.8	0.428	
			CNO	0.0208	0.0229	0.91	0.366	
Male	10.119	0.000	CLZ	0.0199	0.0097	2.05	0.045	*
			DMC	−0.0096	0.0092	−1.04	0.304	
			CNO	0.0554	0.0286	1.94	0.057	
Female	3.727	0.014	CLZ	0.137	0.0541	2.53	0.013	*
			DMC	0.0954	0.0881	1.08	0.282	
			CNO	0.0864	0.1085	0.8	0.428	
**LVEF**								
Male + female	7.152	0.000	CLZ	−0.007	0.004	−1.8	0.077	
			DMC	−0.007	0.006	−1.25	0.218	
			CNO	−0.004	0.006	−0.62	0.538	
Male	10.793	0.000	CLZ	−0.004	0.007	−0.64	0.590	
			DMC	−0.006	0.007	−0.86	0.481	
			CNO	−0.03	0.009	−3.32	0.080	
Female	2.644	0.083	CLZ	−0.011	0.022	−0.51	0.621	
			DMC	−0.004	0.015	−0.26	0.800	
			CNO	0.003	0.01	0.25	0.809	

* *p* < 0.05, β: randomly varying coefficients.

**Table 6:** Impacts of daily dose and exposure duration of clozapine, CPK, eGFR, and monocytes on NT-proBNP and LVEF using MRA.

NT-proBNP	Adjusted R2	F	*p*	Factor	b	SE	T	*p*	
ALL	0.027	2.064	0.073	DU	0.0184	0.0122	1.51	0.133	
				Dose	0.0244	0.0131	1.86	0.065	
				Mono	0.0112	0.0119	0.94	0.348	
				eGFR	−0.0572	0.0957	−0.60	0.551	
				CPK	0.0165	0.0240	0.69	0.493	
JPN	0.086	3.094	0.013	DU	0.1902	0.0737	2.58	0.012	*
				Dose	0.0014	0.0155	0.09	0.927	
				Mono	0.0300	0.0149	2.01	0.048	*
				eGFR	0.0183	0.1381	0.13	0.895	
				CPK	0.0023	0.0275	0.08	0.933	
CHN	0.028	2.40	0.047	DU	0.0244	0.0110	2.22	0.030	*
				Dose	−0.0266	0.0215	−1.24	0.219	
				Mono	−0.0165	0.0119	−1.39	0.170	
				eGFR	−0.1034	0.1046	−0.99	0.327	
				CPK	0.0472	0.0493	0.96	0.342	
Male	−0.007	1.284	0.278	DU	0.0123	0.0167	0.74	0.462	
				Dose	0.0080	0.0161	0.50	0.622	
				Mono	0.0169	0.0147	1.15	0.253	
				eGFR	−0.0524	0.1182	−0.44	0.659	
				CPK	0.0296	0.0273	1.08	0.281	
Female	0.071	1.785	0.131	DU	0.0216	0.0209	1.03	0.305	
				Dose	0.0528	0.0322	1.64	0.107	
				Mono	0.0159	0.0224	0.71	0.480	
				eGFR	0.1164	0.1888	0.62	0.540	
				CPK	−0.0003	0.1115	0.00	0.998	
LVEF	Adjusted R2	F	*p*	factor	b	SE	T	*p*	
ALL	0.045	2.187	0.057	DU	0.0016	0.0023	0.69	0.489	
				Dose	−0.0089	0.0048	−1.86	0.066	
				Mono	0.0004	0.0021	0.21	0.831	
				eGFR	−0.0259	0.0207	−1.25	0.213	
				CPK	−0.0083	0.0054	−1.54	0.126	
JPN	−0.090	0.336	0.888	DU	−0.0062	0.0276	−0.22	0.824	
				Dose	−0.0056	0.0068	−0.82	0.415	
				Mono	0.0014	0.0038	0.37	0.716	
				eGFR	−0.0059	0.0513	−0.11	0.910	
				CPK	−0.0053	0.0089	−0.59	0.556	
CHN	−0.005	1.083	0.372	DU	−0.0005	0.0022	−0.21	0.836	
				Dose	−0.0023	0.0039	−0.59	0.555	
				Mono	0.0015	0.0023	0.67	0.503	
				eGFR	−0.0294	0.0218	−1.35	0.181	
				CPK	−0.0107	0.0061	−1.74	0.083	
Male	0.046	2.113	0.069	DU	0.0071	0.0037	1.90	0.062	
				Dose	−0.0058	0.0036	−1.62	0.108	
				Mono	0.0021	0.0029	0.74	0.462	
				eGFR	−0.0053	0.0264	−0.20	0.842	
				CPK	−0.0076	0.0078	−0.96	0.337	
Female	0.067	1.533	0.189	DU	−0.0076	0.0047	−1.61	0.111	
				Dose	−0.0145	0.0080	−1.82	0.078	
				Mono	−0.0031	0.0039	−0.79	0.430	
				eGFR	−0.0690	0.0471	−1.47	0.147	
				CPK	0.0078	0.0187	0.42	0.679	

* *p* < 0.05.

**Table 7:** Fixed effects of plasma levels, daily dose, and exposure duration of clozapine on monocyte counts (Japanese cases analyzed by fixed effects of hierarchical linear regression analysis with robust standard error).

Sex	F	*p*	Factor	β	SE	T	*p*	
Male + female	5.688	0.000	Duration	1.029	0.107	9.63	0.011	*
			Dose	0.007	0.008	0.89	0.470	
			CLZL	0.104	0.024	4.31	0.049	*
Male	6.015	0.000	Duration	1.482	0.151	9.80	0.010	*
			Dose	−0.009	0.017	−0.53	0.647	
			CLZL	0.076	0.017	4.44	0.047	*
Female	4.779	0.007	Duration	1.590	0.115	13.79	0.005	**
			Dose	0.108	0.284	0.38	0.707	
			CLZL	0.179	0.070	2.55	0.013	*

** *p* < 0.01, * *p* < 0.05.

**Table 8:** Random effects of daily dose and exposure duration of clozapine on monocytes counts using MRA.

Monocytes	Adjusted R^2^	F	*p*	Factor	β	SE	T	*p*	
ALL	0.057	8.822	0.000	Duration	−0.062	0.068	−0.91	0.363	
				Dose	0.275	0.075	3.65	0.000	**
JPN	0.073	4.967	0.009	Duration	−0.144	0.589	−0.24	0.808	
				Dose	0.390	0.154	2.53	0.013	*
CHN	−0.006	0.593	0.554	Duration	−0.047	0.076	−0.62	0.536	
				Dose	0.075	0.110	0.68	0.496	
Male	0.042	5.218	0.006	Duration	−0.027	0.086	−0.32	0.751	
				Dose	0.261	0.092	2.83	0.005	*
Female	0.050	4.041	0.020	Duration	−0.100	0.097	−1.03	0.307	
				Dose	0.258	0.129	1.99	0.049	*

** *p* < 0.01, * *p* < 0.05.

## Data Availability

The original contributions presented in this study are included in the article/Supplementary Materials. Further inquiries can be directed to Prof Motohiro Okada.

## Supplementary Materials

The following supporting information can be downloaded at: https://www.mdpi.com/article/10.3390/medsci14020306/s1. Supplementary Table S1: List of concomitant non-antipsychotic medications. Supplementary Figure S1: Flow diagram of study cohort. Supplementary Table S2: Cases indicating abnormalities of NT-proBNP, BNP or LVEF. Supplementary Figure S2: Impacts of clozapine exposure duration within 100 months on NT-proBNP in Japan and China.

## Abbreviations

The following abbreviations are used in this manuscript:

BNP	Brain natriuretic peptide
CNO	Clozapine-N-oxide
DMC	Desmethyl-clozapine
EF	Ejection fraction
eGFR	Estimated glomerular filtration rate
HF	Heart failure
HFpEF	Heart failure with preserved ejection fraction
HFrEF	Heart failure with reduced ejection fraction
HLM	Hierarchical linear regression model with robust standard error
LMM	Linear mixed model for repeated measures
LVEF	Left ventricular ejection fraction
MRA	Multiple regression analysis
PP2A	Protein phosphatase 2A
