# Supplementary material for: Risk of Long-Term Clozapine Medication over Decades for Cardiac Adverse Events Including Heart Failure and Its Pathophysiology: A Japan and China Retrospective Cohort Analysis
Source: Med Sci (Basel). 2026 Jun 11;14(2):306. doi: 10.3390/medsci14020306 (PMC13302769; doi:10.3390/medsci14020306)
Supplement: Supplementary file 1 [file medsci-14-00306-s001.zip › medsci-4312402-supplementary.pdf]

Supplementary Table S1. List of concomitant non-antipsychotic medications

Medications	Case numbers	Major metabolic enzymes					
Elobixibat	76		CYP3A4				
Amlodipine	11		CYP3A4				
Valbenazine	9		CYP3A4			CYP3A5	
Bilastine	9		CYP3A4				
Tamsulosin	7		CYP3A4		CYP2D6		
Domperidone	6		CYP3A4				
Lansoprazole	5		CYP3A4	CYP2C19			
Mosapride	4		CYP3A4				
Levocetirizine	4		CYP3A4				
Teneligliptin	3		CYP3A4				
Bisoprolol	3		CYP3A4		CYP2D6		
Olopatadine	1		CYP3A4				
Silodosin	1		CYP3A4				
Mitiglinide	1		CYP3A4				CYP2C9
Spironolactone	1		CYP3A4				
Carvedilol	1				CYP2D6		CYP2C9
Propranolol	2	CYP1A2		CYP2C19	CYP2D6		

Supplementary Figure S1: Flow diagram of study cohort.

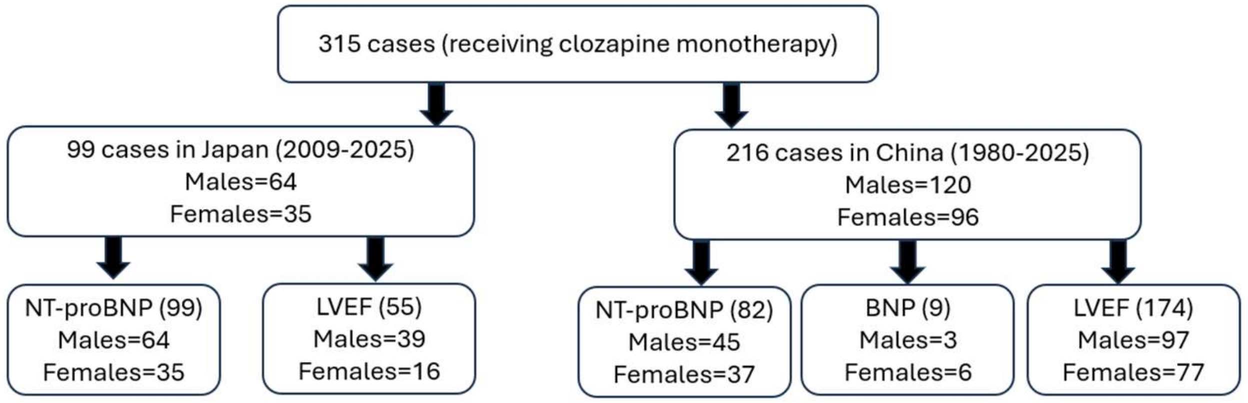

Supplementary Table S2. Cases indicating abnormalities of NT-proBNP, BNP or LVEF

Country	NT-proBNP (<125)	BNP (<35)	LVEF (>50)	Duration (month)	Age (year)	Sex	CLZ Dose (mg/day)	eGFR (>90)	HbA1c (<6.5)	TG (<150)	T-Cho (<220)	CLZ (350–600)	DCLZ (100–300)	CNO	Complication	
Japan	1240		36	33	28	M	225	89.1	5.7	254	208				Clozapine-induced cardiomyopathy	Enalapril, Bisoprolol
	170		51	64	37	M	500	91.3	5.1	49	96.0	646	569	124		Valbenazine
	131		59	57	33	F	125	100.8	5.9	237	203	93	78	17	-	
	733		35	11	42	M	400	85.8	5.8	73	168	729	444	202	Old myocardial infarction	Dapagliflozin
	458		59	17	64	M	250	52.3	5.2	113	125	437	533	160	Old myocardial infarction	Carvedilol, Edoxaban, Spironolactone
	170		46	23	59	F	350	53.6	5.7	250	212	162	212	37	Ileus	Lubiprostone, Elobixibat
	49.7		35	8	21	M	200	131.2	5.2	101	198	287	234	59	Ileus	Lubiprostone, Elobixibat
	552		69	2	48	M	50	24.9	5.3	79	144	205	83	52	renal dysfunction	
	27		45	86	49	M	400	93.7	5.8	123	203	261	224	95	cardiomyopathy	Macrogol, Elobixibat
China	142			204	61	M	225	119.0	4	97	139	-	-	-	-	
	149			326	65	F	300	78.3	5.2	140	151	-	-	-	-	
	129		71	149	28	M	300	118.2	5.3	54	143	-	-	-	-	
	433		66	241	54	M	250	79.7	5.6	119	184	-	-	-	-	
		250		2	70	F	200	51.1	5.4	96	144	-	-	-	-	
		298		20	70	F	125	48.3	5.4	74	138	-	-	-	-	
		42.3		278	63	F	100	82.5	5.6	101	166	-	-	-	-	
		47.2		88	66	F	125	69.1	5.9	83	138	-	-	-	-	
	127		73	15	70	F	75	46.0	6.5	304	232	-	-	-	diabetes mellitus	
	127		71	362.6	57	F	250	94.04	7.5	373	152	-	-	-	diabetes mellitus	
	1832		64	19.9	62	M	225	71.04	8.2	45	169	-	-	-	diabetes mellitus	
	151		62	483	59	M	250	107.57	8.4	161	213	-	-	-	diabetes mellitus	
	219		64	399	78	M	50	26.82	6.3	110	191	-	-	-	renal dysfunction	

Supplementary Figure S2: Impacts of clozapine exposure duration within 100 months on NT-proBNP in Japan and China.

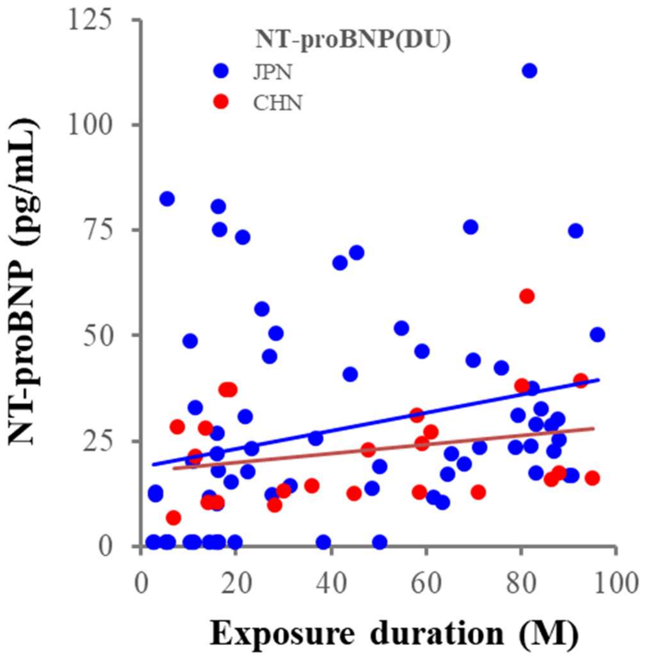

Ordinates indicate NT-proBNP level (pg/mL), and abscissas indicate duration of clozapine exposure (months).

Blue and red circles indicate Japanese and China cases, respectively. Lines indicate the trends detected by ANCOVA.

$F_{region}(1,95)=0.1$ ($p>0.1$), $F_{duration}(1,95)=6.6$ ($p<0.05$), $F_{region*duration}(1,95)=0.2$ ($p>0.1$).
